# Stress Hyperglycaemia Indicates Embolus Size and Localization in Patients with Acute Pulmonary Embolism

**DOI:** 10.1155/2020/3606757

**Published:** 2020-07-08

**Authors:** Velimir Altabas, Lucija Pukec, Stella Mlinarić, Hrvoje Pintarić, Aljoša Šikić

**Affiliations:** ^1^School of Medicine, University of Zagreb, Zagreb, Croatia; ^2^Clinic for Internal Medicine, Department of Endocrinology, Diabetes and Metabolic Diseases, Sestre Milosrdnice University Hospital Center, Zagreb, Croatia; ^3^Emergency Department, Sestre Milosrdnice University Hospital Center, Zagreb, Croatia

## Abstract

**Objective:**

Acute pulmonary embolism is a life-threatening form of venous thromboembolism often causing stress hyperglycaemia. The aim of this study was to determine the prognostic value of stress hyperglycaemia in acute pulmonary embolism, providing new insights into the presumed embolus size and localization, clinical parameters (Pulmonary Embolism Severity Index, PESI), and in-hospital mortality. *Design and Methods*. Among a total of 95,454 patients referred to the Emergency Department of the Sestre Milosrdnice University Hospital Centre between 2014 and 2016, all patients with acute pulmonary embolism were included into this observational cohort study. The study group consisted of 190 patients aged 25–96. Relevant patient history, clinical data, and laboratory findings were collected during the entire hospitalization period. Data were analyzed for the entire group of patients, as well as separately for patients without diabetes, using the Fisher exact test and logistic regression.

**Results:**

Analysis of embolus localization as an indirect parameter of embolus size showed that patients with stress hyperglycaemia more often had emboli located in proximal parts of the pulmonary circulation (i.e., main artery or lobar branches) (*p* < 0.05). Furthermore, stress hyperglycaemia correlated with PESI score and diabetes (*p* < 0.05) in the entire patient group. Stress hyperglycaemia showed independent association with in-hospital mortality in patients (*p* < 0.05).

**Conclusion:**

Stress hyperglycaemia in patients with acute pulmonary embolism is associated with embolus localization in larger arteries of the pulmonary circulation and higher PESI score and therefore could serve as an independent in-hospital mortality predictor.

## 1. Introduction

Acute pulmonary embolism (APE) is a life-threatening form of venous thromboembolism (VTE) often associated with stress hyperglycaemia (SH). Risk factors for APE are divided into two groups, environmental and genetic. Patient' history of deep vein thrombosis (DVT), malignancy, patient age, neurological disease, trauma or fracture, immobilization, and major surgery are closely related to the occurrence of VTE, including pulmonary embolism (PE) [1–3].

Clinical presentation of APE is diverse and does not always correlate with radiographic findings; however, multislice computed tomography angiography (MSCTA) is the gold standard in diagnosing PE with a sensitivity of 83–100% and specificity of 89–96%. Other procedures, such as Pulmonary Embolism Severity Index (PESI) scoring, are used to estimate clinical outcomes [4]. PESI is a prognostic model for APE that includes 11 clinical criteria that predict a 30-day patient mortality [5].

SH refers to transient elevated plasma glucose (PG), most commonly detected during hospital admission. It is a frequent laboratory finding in severe acute medical and surgical conditions in patients without diabetes mellitus (DM), although SH may also occur in patients with formerly well-controlled DM [6–8]. There is a prevalent misconception that SH equals diabetes, but it has to be stressed that these are two separate yet, similar conditions. Stress hyperglycemia occurs secondary to an acute disease and will disappear over time in the majority of patients, while diabetes is a longstanding condition resulting in chronic complications.

The onset mechanisms of hyperglycaemia in DM and SH only partially overlap. The pathophysiology of SH is based on the neuroendocrine and inflammatory response to acute stressful events. Activation of the hypothalamus-pituitary-adrenal axis and the sympathetic nervous system results in increased secretion of counterregulatory hormones (i.e., cortisol, glucagon, adrenaline, noradrenaline, and growth hormone) that leads to changes in carbohydrate metabolism [8]. As part of the stress response, mediators of inflammation are secreted, reducing glucose utilization in peripheral tissues and stimulating the release of counterregulatory hormones, which enhances hyperglycaemia, cytokine secretion, inflammatory response, and oxidative stress and closes the vicious cycle [6]. However, hyperglycaemia in diabetes is mostly caused by defects in insulin secretion, insulin resistance, and functional disruptions of various organs (e.g., the brain, gut, and kidney) [9].

Previous research has demonstrated the occurrence of SH in myocardial infarction, stroke, trauma, burns, sepsis, postoperative, and in APE [7, 8, 10–12], but due to nonharmonized criteria for SH, this condition is still not well understood. For example, there is no universally accepted threshold for the blood glucose level to define SH, which complicates calculating the prevalence of SH and its consequences [13, 14]. According to a systematic literature review by Olariu et al., SH has been consistently associated with poor disease outcomes [13]. In APE, elevated admission PG has been shown to be an independent risk factor for a 30-day mortality [7]. A few studies have shown that for patients with acute myocardial infarction and the patients with SH without previous diagnosis of DM have more severe outcomes than those with DM [15].

The aim of this study was to investigate correlations between SH, embolus size and localization, clinical parameters, and in-hospital mortality in APE.

To our best knowledge, there is no evidence in current literature about SH indicating specific radiological findings that may impact prognostic scores and in-hospital mortality in patients with APE.

## 2. Materials and Methods

This observational cohort study was conducted in concordance with the Declaration of Helsinki as revised in 2008, and it was approved by the ethical boards of the Sestre Milosrdnice University Hospital Centre and of the School of Medicine at the University in Zagreb.

Between January 01, 2014 and December 31, 2016, there were 95,454 patients admitted to the Emergency Department at Sestre Milosrdnice University Hospital Centre in Zagreb. All patients underwent standard admission protocols (i.e., medical history, physical examination, and basic laboratory tests, including PG and electrocardiogram). All patients with suspected APE underwent MSCTA to confirm the diagnosis. Among them, 253 patients, aged 25–96, were hospitalized for acute PE in Sestre Milosrdnice University Hospital Centre in Zagreb and were recruited into the study. Relevant patient information was collected from electronic records.

Patients whose symptoms lasted for 7or more days (*n* = 48), those who did not get MSCTA confirmation because of sudden death (*n* = 3), and those with radiographic signs of chronic PE (*n* = 2) were excluded from the study. Based on radiological findings, localization of the embolus was used to indirectly define the approximate embolus size, assuming that large emboli cannot reach vessels with a smaller diameter without dissolution. Emboli found in the main or in the lobar branches of pulmonary arteries were considered as large, while small emboli were those found in segmental or subsegmental branches. Final PESI score was assigned by summing points for each characteristic as in the original PESI study [16]. To calculate the PESI score, data on age, sex, heart failure, chronic lung disease, malignancy, mental status, systolic pressure, heart rate, respiratory rate, body temperature, and oxygen saturation at admission were collected. According to age, patients were divided into younger patient group (<65 years old) and older patient group (65 years and older), as in the original PESI study. In statistics, PESI classes 1 and 2 were considered low class, while classes 3, 4, and 5 were considered high class. Patients who did not have all anamnestic or clinical data necessary for PESI scoring were excluded (*n* = 10).

The study sample consisted of 190 patients, 154 nondiabetics and 36 diabetics.

PG values used in this study were the earliest ones measured in the emergency department. The cut-off value used for SH in this study was ≥7.8 mmol/L, as used by the American Diabetes Association for elevated blood glucose in chronic conditions such as prediabetes and diabetes.

Because of chronic hyperglycaemia in patients with DM with fair blood glucose control, criteria for SH should be different for this subgroup of diabetic patients, and therefore, data on diabetic patients should be interpreted with more caution.

Because of the small sample size, metabolic changes, possible previous fair blood glucose control, and therapy interference, data on patients with DM were not specifically analyzed.

Finally, data on in-hospital mortality were collected.

Statistical analysis was done using descriptive statistics, the Fisher exact test (FET) for the univariate analysis, and logistic regression for the multivariate analysis. Significance level used in tests was 0.05. Analysis was done using MS Office 2013 and MedCalc® 19.1.7 (MedCalc, Ostend, Belgium).

## 3. Results

Patient characteristics are shown in Table 1.

In this study, the statistical analysis was done for the entire patient group (*n* = 190), along with a subgroup of patients without DM (*n* = 154). The data were compared to point out the differences depending on inclusion or exclusion of diabetic patients.

According to the Fisher exact test for both the nondiabetics and the entire patient group, SH was shown to be associated with the PESI class (Table 2). This association remained significant after logistic regression in the group of patients without diabetes (*p*=0.0075). In the entire patient group, SH was additionally independently associated with DM (*p*=0.0004) and embolus localization (*p*=0.0248). After logistic regression, diabetes (*p*=0.0009), embolus location (*p*=0.0161), and PESI class (*p*=0.0048) showed independent association with SH.

A total of 25 patients died during hospitalization, 20 diabetics and 5 nondiabetics.

SH and patient history of malignancy were associated with in-hospital mortality by using the Fisher exact test in patients without diabetes. In the entire patient group, age group, SH, and PESI class were associated with mortality after FET.

After logistic regression, independent association with in-hospital mortality in patients without DM was shown for SH (*p*=0.0084) and patient history of malignancy (*p*=0.0230). In the entire patient group, SH showed significant association with in-hospital mortality (*p*=0.0495) after logistic regression (Table 3).

## 4. Discussion

In general, the main problem in SH studies is defining cut-off values. Among different studies, these values range from 6.1 mmol/L to 11.1 mmol/L, which decreases reliability and complicates comparison of results. It was recommended that guidelines about SH values for this clinical entity should be made, but they still do not exist. Furthermore, in contrast to a vast quantity of research regarding cardiovascular outcomes related to stress hyperglycemia, there is only little evidence about SH and acute PE [7, 17–19].

The prevalence of SH in acute PE in this study (46.84%) is higher than the prevalence reported by Scherz et al. (33.58%) for the 7.8 mmol/L cut-off value [7]. The higher value is probably due to the more advanced age and a higher prevalence of diabetes among patients in this study.

In the entire group of patients, logistic regression showed that DM and PESI score are risk factors for developing SH, confirming results from previous studies [7, 20].

SH correlated well with PESI scores, which is a prognostic indicator combining several clinical parameters.

Furthermore, in the entire study population, the embolus localization as an indirect measure of embolus size was also shown to be an independent risk factor for developing SH. These findings fit perfectly into the thesis that more profound stress is due to more massive embolization and more elevated admission glycaemia compared to embolization of more distal smaller vessels. However, further investigation will be needed to prove this concept in patients without diabetes.

The PESI model, commonly used to predict the 30-day mortality, showed no significance after logistic regression with in-hospital mortality for any patient group. In contrast to insignificant PESI statistic outcomes, SH showed independent association with in-hospital mortality in both patient groups, indicating that it could be used as a less-complicated prognostic factor for in-hospital outcomes.

Similar data were described in a recent study comparing the PESI score with a composite biomarker prognostic model that included admission glycaemia [17] and in a study analyzing the separate impacts of diabetes and SH on APE-related mortality [18].

There are some limits to this study that need to be clarified. First, as in the original PESI study, mostly categorical data (i.e., binary data for presence or absence of hypotension or tachycardia) instead of qualitative data (i.e., exact numbers for blood pressure and heart rate) were used [5]. Second, patients who have had emboli in several different branches of pulmonary artery were referred to the localization group according to the biggest branch blocked. In this way, the total size of the PE could have been overseen, especially in patients with a large number of smaller vessels blocked. Third, patients with DM were not statistically evaluated because of the small sample size and specific issues regarding their glucose metabolism (e.g., chronic hyperglycaemia and concomitant antidiabetic medication affecting SH).

## 5. Conclusion

SH is common in patients with APE like in a variety of other acute medical and surgical conditions. In general, SH is independently associated with embolus localization on MSCTA, PESI score, and in-hospital mortality.

Considering PG is a simple test that can be measured quickly and provides additional information about clinical outcomes; it should not be overlooked in the diagnosis and prognosis estimation of many acute diseases, including APE.

## Figures and Tables

**Table 1:** Patient characteristics.

	All patients (190)	Nondiabetics (154)	Diabetics (36)
Demographics			
Age (median, range)	76 (82–64)	75.5 (82–62)	79 (83–68.5)
Age (<65 years)	49 (25.79%)	43 (27.92%)	6 (16.67%)
Men, *n* (%)	70 (36.84%)	57 (37.01)	13 (36.11)

Medical history (*n*, %)			
Hypertension	109 (57.37)	79 (51.30)	30 (83.33)
Diabetes mellitus	36 (18.95)	—	—
Smoking	35 (18.42)	31 (20.13)	4 (11.11)
History of thromboembolism	29 (15.26)	24 (15.58)	5 (13.89)
Immobility	32 (16.84)	23 (14.94)	9 (25.00)
History of malignancy	51 (26.84)	41 (26.62)	10 (27.78)
History of heart failure	8 (4.21)	6 (3.90)	2 (5.56)
History of COPD	25 (13.15)	22 (14.29)	3 (8.33)

Clinical admission data (*n*, %)			
BMI (kg/m^2^)	28.39 (±5.88)	28.03 (±5.77)	27.88 (±9.98)
Stress hyperglycaemia	89 (46.84)	62 (40.26)	27 (75.00)
Respiration ≥ 30/min	22 (11.58)	17 (11.04)	5 (13.89)
Saturation O_2_ < 90%	82 (43.16)	63 (40.91)	19 (52.78)
Axillary temperature < 36°C	1 (0.01)	1 (0.64)	0
Altered mental status	20 (10.53)	17 (11.04)	3 (8.33)

Embolus localization			
Main and lobar branches	137 (72.11)	111 (72.08)	26 (72.22)
Segmental and subsegmental branches	53 (27.89)	43 (27.92)	10 (27.78)

PESI class (*n*, %)			
Low	55 (28.95)	49 (31.82)	6 (16.67)
High	135 (71.05)	105 (68.18)	30 (83.33)

**Table 2:** Association of stress hyperglycaemia with patient characteristics.

	FET (univariate analysis)		Logistic regression (multivariate analysis)	
	*p*	Odds ratio (95% CI)	*p*	Odds ratio (95% CI)
Patients without DM (154)				
Sex	0.2336	1.5926 (0.806–3.1467)	—	—
Age	0.0669	2.1175 (0.9862–4.5464)	—	—
Diabetes mellitus	—		—	—
Thromboembolic history	1.0000	1.0714 (0.4426–2.5938)	—	—
Malignancy	0.3601	1.4059 (0.6831–2.8936)	—	—
Embolus localization	0.1434	1.8238 (0.8607–3.8647)	—	—
PESI class	**0**.**0022**	2.8700 (1.4639–5.6265)	**0**.**0075**	**2**.**8030 (1**.**3169**–**5**.**9662)**

All patients (190)				
Sex	0.2925	1.4138 (0.7797–2.5637)	—	—
Age	0.0670	1.9642 (1.0005–3.8561)	—	—
Diabetes mellitus	**0**.**0004**	**4**.**4516 (1**.**9601**–**10**.**1101)**	**0**.**0009**	**4**.**2590 (1**.**8136**–**10**.**0015)**
Thromboembolic history	0.6867	1.5297 (0.5708–2.7799)	0.6829	—
Malignancy	0.1926	1.5597 (0.8165–2.9680)	0.5244	—
Embolus localization	**0**.**0248**	**2**.**0918 (1**.**0812**–**4**.**0468)**	**0**.**0161**	**2**.**3844 (1**.**1752**–**4**.**8380)**
PESI class	**0**.**0284**	**2**.**0918 (1**.**0812**–**4**.**0468)**	**0**.**0048**	**2**.**7514 (1**.**3610**–**5**.**5625)**

FET is the Fisher exact test; logistic regression; and significant *p* values are marked in bold.

**Table 3:** Association of in-hospital mortality and patient characteristics.

	FET (univariate analysis)		Logistic regression (multivariate analysis)	
	*p*	Odds ratio (95% CI)	*p*	Odds ratio (95% CI)
Patients without DM (154)				
Sex	0.2204	0.5402 (0.2098–1.3908)	—	—
Age	0.0637	3.9677 (0.8797–17.8963)	—	—
Diabetes mellitus	—		—	—
Thromboembolic history	0.7412	0.5657 (0.1224–2.6142)	—	—
Malignancy	**0**.**0267**		**0**.**0230**	**3**.**1751 (1**.**1729**–**8**.**5955)**
Embolus localization	0.7946	0.8900 (0.3182–2.4897)	—	—
SH	**0**.**0061**	**4**.**1806 (1**.**5082**–**11**.**5876)**	**0**.**0084**	**4**.**0360 (1**.**4303**–**11**.**3889)**
PESI class	0.0729	3.9677 (0.8797–17.8963)		—

All patients (190)				
Sex	0.5056	0.7084 (0.3023–1.6598)	—	—
Age	**0**.**0028**	**4**.**5805 (1**.**0386**–**20**.**2016)**	0.1342	1.0397 (0.9880–1.0941)
Diabetes mellitus	1.0000	1.0806 (0.3763–3.1035)	—	—
Thromboembolic history	0.7722	0.7290 (0.2033–2.6140)	—	—
Malignancy	0.0951	2.4554 (1.0325–5.8388)	—	—
Embolus localization	0.8118	1.2613 (0.4743–3.344)	—	—
SH	**0**.**0092**	**3**.**4044 (1**.**3488**–**8**.**5928)**	**0**.**0495**	**2**.**5974 (1**.**0020**–**6**.**7329)**
PESI class	**0**.**0002**	**25**.**6154 (1**.**5308**–**428**.**6366)**	0.9968	Indeterminate

FET is the Fisher exact test; logistic regression; and significant *p* values are marked in bold.

## Data Availability

The data used to support the findings of this study are available from the corresponding author upon request.
